# Methanolic Extract of* Clinacanthus nutans* Exerts Antinociceptive Activity via the Opioid/Nitric Oxide-Mediated, but cGMP-Independent, Pathways

**DOI:** 10.1155/2016/1494981

**Published:** 2016-04-13

**Authors:** Mohammad Hafiz Abdul Rahim, Zainul Amiruddin Zakaria, Mohd Hijaz Mohd Sani, Maizatul Hasyima Omar, Yusnita Yakob, Manraj Singh Cheema, Siew Mooi Ching, Zuraini Ahmad, Arifah Abdul Kadir

**Affiliations:** ^1^Department of Biomedical Science, Faculty of Medicine and Health Science, Universiti Putra Malaysia, 43400 Serdang, Selangor, Malaysia; ^2^Integrative Pharmacogenomics Institute (iPROMISE), Faculty of Pharmacy, Universiti Teknologi MARA (UiTM), Puncak Alam Campus, Level 7, FF3, 42300 Bandar Puncak Alam, Selangor, Malaysia; ^3^Phytochemistry Unit, Herbal Medicine Research Centre, Institute for Medical Research, Jalan Pahang, 50588 Kuala Lumpur, Malaysia; ^4^Molecular Diagnostics and Protein Unit, Specialised Diagnostics Centre, Institute for Medical Research, Jalan Pahang, 50588 Kuala Lumpur, Malaysia; ^5^Department of Family Medicine, Faculty of Medicine and Health Science, Universiti Putra Malaysia, 43400 Serdang, Selangor, Malaysia; ^6^Department of Veterinary Pre-Clinical Sciences, Faculty of Veterinary Sciences, Universiti Putra Malaysia, 43400 Serdang, Selangor, Malaysia

## Abstract

The objectives of the present study were to determine the mechanisms of antinociceptive effect of methanol extract of* Clinacanthus nutans* (Acanthaceae) leaves (MECN) using various animal nociceptive models. The antinociceptive activity of orally administered 10% DMSO, 100 mg/kg acetylsalicylic acid (ASA), 5 mg/kg morphine, or MECN (100, 250, and 500 mg/kg) was determined using the acetic acid-induced abdominal constriction (ACT), formalin-induced paw licking (FT), and hot plate tests (HPT). The role of opioid and nitric oxide/cyclic guanosine monophosphate (NO/cGMP) systems was also investigated. The results showed that MECN produced a significant (*p* < 0.05) antinociceptive response in all nociceptive models with the recorded ED_50_ value of 279.3 mg/kg for the ACT, while, for the early and late phases of the FT, the value was >500 mg/kg or 227.7 mg/kg, respectively. This antinociceptive activity was fully antagonized by naloxone (a nonselective opioid antagonist) but was partially reversed by l-arginine (l-arg; a nitric oxide [NO] precursor), N*ω*-nitro-l-arginine methyl ester hydrochloride (l-NAME; an NO synthase inhibitor), or their combinations thereof. In contrast, 1H-[1,2,4]oxadiazole[4,3-a]quinoxalin-1-one (ODQ; a soluble guanylyl cyclase inhibitor) enhanced the extract's antinociception. UHPLC analysis revealed the presence of several flavonoid-based compounds with antinociceptive action. In conclusion, MECN exerted the peripherally and centrally mediated antinociceptive activity via the modulation of the opioid/NO-mediated, but cGMP-independent, systems.

## 1. Introduction

Opioids, such as morphine, and nonsteroidal anti-inflammatory drugs (NSAIDs), such as acetylsalicylic acid, are universally used for the treatment of pain. Although treatments for pain have seen rapid progression, particularly in the field of analgesic drug development, their clinical efficacy and tolerability are often surpassed by the accompanied unwanted adverse effects [1]. Therefore, there is a need to look for an alternative approach to treat pain that has fewer or, possibly, no side effects. Drugs derived from natural sources, especially plants, are vital for the treatment of numerous diseases [2]. The exploration and investigation of plants utilized as pain-relieving agents in traditional ethnomedicine is one of the useful and reasonable strategies in the search for new drugs [3, 4].

Treatment of pain involved the usage of opioids and nonsteroidal anti-inflammatory drugs, and, despite their effectiveness in curing pain, prolonged usage of these classes of drugs has been associated with various unwanted side effects [5]. The risk from NSAID use involves increased GI bleeding and ulceration, increased potential for myocardial infarction, stroke, and Stevens-Johnson syndrome. Opioids, used for moderate-to-severe pain, provide excellent pain relief and are easier to metabolize but have the unwanted effects of sedation, nausea, confusion, and delirium [6]. Other than that, certain types of pain like cancer-related pain are not effectively treated with conventional drugs; thus, patients suffering from this type of pain will seek for alternative treatment [5]. Nowadays the number of patients that are using herbal remedies and complementary and alternative medicine for treatment of pain is growing rapidly [7]. Over the last 20 years, Americans have sought a more “natural” or “holistic” approach to treatment of medical problems in general and pain in particular. Americans spend billions of dollars annually to find a holistic treatment with effective pain relief and few side effects, on complementary and alternative medicine, including herbal therapies [8]. Such increase in popularity and use of CAM by the general public strongly demands that health care professionals have the knowledge to assess, intervene, and advise patients on effective and safe CAM practices [9, 10].

One of the medicinal plants that have gained attention among the scientists is* Clinacanthus nutans* (Burm. f.) Lindau, a plant belonging to the family Acanthaceae. Locally known as “*Belalai Gajah*,” it is a shrub native to the tropical Southeast Asian countries. The fresh leaves are consumed raw as vegetables and mixed with juices and can be used to brew drinks; the dried leaves can be steeped in hot water and served as herbal tea [11]. In Indonesia, Malaysia, and Thailand in particular, the plant is traditionally used in the treatment of skins rashes, insect and snake bites, mental stress, diabetes, rheumatoid arthritis, fever, dysentery, burns, scalds, diarrhea, and herpes skins infections [11]. Scientifically, extracts of* C. nutans* have been shown to exert antibacterial [12], anti-inflammatory [13], antiherpes [14, 15], antioxidant [16], antiproliferative [17], cytotoxic, and antimutagenic [18] activities and demonstrated to affect the immune response when studied* in vivo* (mice) [19] or* in vitro* (human cells) [20]. Moreover, the plant has also been developed into oral-based agent for the treatment of recurrent aphthous stomatitis [21] while the oral toxicity study revealed that* C. nutans* is safe for consumption [22].

Various chemical constituents (i.e., stigmasterol, lupeol, *β*-sitosterol, betulin, vitexin, isovitexin, schaftoside, isomollupentin-7-O-*β*-glucopyranoside, orientin, isoorientin, sulfur-containing glucosides, glycoglycerolipids, and monoacylmonogalactosylglycerol) have been isolated and identified from* C. nutans* [11]. However, the bioactivity of some of these compounds still remains to be elucidated. Additionally the presence of* n*-pentadecanol, eicosane, 1-nonadecene, heptadecane, dibutyl phthalate,* n*-tetracosanol-1, heneicosane, behenic alcohol, 1-heptacosanol, 1,2-benzenedicarboxylic acid, mono(2-ethylhexyl) ester, nonadecyl heptafluorobutyrate, eicosanoyl trifluoroacetate, 1,2-benzenedicarboxylic acid, dinonyl ester, phthalic acid, and dodecyl nonyl ester was reported in the chloroform extract of* C. nutans* leaves [19]. Several other compounds have also been identified and further demonstrated to have some degrees of bioactivity. For example, three types of phaeophytins, namely, 13^2^-hydroxy-(13^2^-*R*)-phaeophytin b, 13^2^-hydroxy-(13^2^-*S*)-phaeophytin a, and 13^2^-hydroxy-(13^2^-*R*)-phaeophytin, have been identified from the chloroform extract of* C. nutans* leaves and were reported to exhibit anti-herpes simplex activity [23]. Despite the various reports on pharmacological activity of* C. nutans*, there has been no study on MECN's antinociceptive activity to date. The proposed antinociceptive study is attributed to finding that* C. nutans* exerts anti-inflammatory activity [14] and contains several classes of phytoconstituents (i.e., flavonoids, saponins, and triterpenes) that are strongly associated with antinociceptive activity [13]. Thus, the present study aimed at determining the antinociceptive activity of methanol extract of* C. nutans* (MECN) and to elucidate the possible mechanisms of antinociception involved.

## 2. Materials and Methods

### 2.1. Plant Material and Extraction

Fresh* C. nutans* leaves were obtained from Clinnthus Enterprise (Kuala Lumpur, Malaysia) in January 2013. Authentication of the plant was made by Dr. Shamsul Khamis, a botanist from the Institute of Bioscience, Universiti Putra Malaysia, Serdang, Selangor, Malaysia, and a voucher specimen (SK 2679/15) has been deposited at the herbarium of the institute. Extraction was carried out according to the method previously described [24]. To obtain the MECN, 250 g of* C. nutans *leaves, which were dried in an oven at 40°C for 1-2 days and ground into powder form using an electric grinder (RT-08; Rong Tsong Precision Technology, Taichung, Taiwan), was soaked in methanol (Fisher Scientific, Loughborough, England) in the ratio of 1 : 20 (w/v) for 72 hours at room temperature. The supernatant was filtered using a steel filter, cotton wool, and Whatman Number 1 filter paper. The residue underwent the same soaking procedures twice. The supernatant collected from each extraction was pooled and evaporated using a vacuum rotary evaporator (Hei-VAP Value; Heidolph, Schwabach, Germany) at 40°C under reduced pressure. These processes yielded approximately 53 g of dried MECN (yield was 21.2% (w/w)), which was then stored at 4°C until it was used.

### 2.2. Phytochemical Screening of MECN

The phytochemical screening of fractions was performed according to the conventional protocols as described by Ikhiri et al. [25].

### 2.3. Chemicals Used in the UHPLC Analysis of MECN

Formic acid, methanol, and LCMS grade acetonitrile were purchased from Merck (Darmstadt, Germany). HPLC grade water was prepared from distilled water using a Milli-Q-system (Millipore, MA, USA) and was used during analytical HPLC analysis. Various pure flavonoid-based standards (HPLC grade) were purchased from Extrasynthese (Lyon, France). All of the other solvents and chemicals used in this study were of analytical grade. Stock and working standards were prepared by dissolving these analytes in 100% methanol. The standard solutions stored at 4°C were stable for at least 3 months.

### 2.4. UHPLC-ESI Profiling of MECN

The UHPLC system was performed on a Dionex 3000 UHPLC system acquired from Thermo Fisher Scientific (USA) that consists of an autosampler equipped with a column oven, a tray compartment cooler, and a binary pump with built-in solvent degasser. Samples (10 *μ*L) were injected and the chromatographic separation was performed on a BEH C18 UHPLC column, 100 mm × 2.5 *μ*m, 1.7 *μ*m (WATERS) at a flow rate of 0.3 mL/min. The mobile phases used were (A) 0.1% formic acid in water and (B) 0.1% formic acid in acetonitrile. The separation was conducted using the following multistep gradient: initial conditions (*t* = 0 min) were 90% A and 10% B with a linear gradient reaching 15% B at *t* = 3 min. The gradient was then increased to 50% B in the next 7 min (*t* = 10 min) and further increased to 90% B for the next 2 min (*t* = 12 min). Finally, the programme was returned to the initial solvent composition at *t* = 17 min for the next analysis.

The UHPLC system was coupled to a Linear Ion Trap Orbitrap mass spectrometer (Q Exactive) from Thermo Fisher Scientific (USA) equipped with an electrospray ionization (ESI) source. The mass detection was performed in a range of 150–1500* m/z*. The ESI source was operated in negative ion mode under the following specific conditions: source voltage: 3.2 kV; sheath gas: 35 arbitrary units; auxiliary gas: 15 arbitrary units; sweep gas: 10 arbitrary units; and capillary temperature: 320°C. Nitrogen (>99.98%) was employed as sheath, auxiliary, and sweep gas. Instrument control and data acquisition were performed with Chameleon 6.8 software and Xcalibur 2.2 software (Thermo Fisher Scientific).

### 2.5. GC-MS Analysis of MECN

GC-MS analysis of MECN was performed using Agilent 7890A (Agilent Technologies) coupled with MSD quadrupole detector 5975 C (Agilent Technologies). Separation of analytes by gas chromatography was carried out using a Hewlett Packard HP-5MS silica capillary column (30 m × 0.25 mm × 0.25 mm). For GC-MS detection, an electron ionization system with ionizing energy of 70 eV was used. Helium gas (99.999%) was used as the carrier gas at constant flow rate 1 mL/min and an injection volume of 1 *μ*L was employed (split ratio of 1 : 10), injector temperature was 250°C, and ion-source temperature was 280°C. The oven temperature was programmed from 100°C (isothermal for 2 min), with an increase of 10°C/min, to 200°C and then 12°C/min to 280°C, ending with a 17 min isothermal at 280°C. Mass spectra were taken at 70 eV, a scan interval of 0.5 sec, and fragments from 45 to 450 Da. Total GC running time was 35.50 min. The relative % amount of each component was calculated by comparing its average peak area to the total areas; software adopted to handle mass spectra and chromatograms was a Turbomass. Interpretation on mass spectrum GC-MS was conducted using the database of National Institute Standard and Technology (NIST) having more than 62,000 patterns. The spectrum of the unknown components were compared with the spectrum of the known components stored in the NIST library.

### 2.6. Experimental Animals

The antinociceptive studies were carried out using either the adult male ICR mice (25–30 g) or Sprague-Dawley rats (150–180 g), which were obtained from the Animal Source Unit, Faculty of Veterinary Medicine, Universiti Putra Malaysia (UPM), Serdang, Malaysia. The animals were kept at room temperature (27 ± 2°C; 70–80% humidity; 12 h light/dark cycle) in the Animal Holding Unit, Faculty of Medicine and Health Science, UPM, for at least 48 h prior to the procedure. Commercial food pellets (Gold Coin Feedmills, Port Klang, Malaysia) and water were supplied* ad libitum*. The animal experimental protocols were in accordance with the current guidelines for the care of laboratory animals and the ethical guidelines for investigations of experimental pain in conscious animals as adopted from Zimmermann [26] and have been approved by the UPM Institutional Animal Care and Use Committee (Ref. Number UPM/IACUC/AUP-R032/2013). The number of animals and intensities of noxious stimuli used were the minimum necessary to demonstrate the consistent effects of the treatments. Experiments were conducted between 0930 and 1830 h to minimize the effects of environmental changes.

### 2.7. Drugs and Chemicals

Acetylsalicylic acid (ASA), morphine hydrochloride, naloxone hydrochloride, l-arginine (l-arg), N*ω*-nitro-l-arginine methyl ester hydrochloride (l-NAME), and 1H-[1,2,4]oxadiazole[4,3-a]quinoxalin-1-one (ODQ) were purchased from Sigma-Aldrich (St. Louis, MO, USA). Formaldehyde was purchased from R & M Chemicals (Essex, England). Acetic acid, dimethyl sulfoxide (DMSO), and methanol were purchased from Fisher Scientific (England). Drugs were dissolved in physiological saline (0.9% (w/v) NaCl). Morphine and ASA were prepared by dissolving in distilled water; MECN was dissolved in 10% DMSO (v/v) in distilled water. Control animals received only solvent vehicle. All drugs, chemicals, and MECN solutions were administered in the volume of 10 mL/kg and were freshly prepared just before use. The MECN doses (100, 250, and 500 mg/kg) used were based on our recent acute and subchronic toxicity studies of MECN (personal communication), which were further supported by the previous oral toxicity studies that reported no toxic or sedative effects at the stated doses [22, 27].

### 2.8. Nociceptive Tests

#### 2.8.1. Acetic Acid-Induced Abdominal Constriction Test

The procedure was conducted as previously described [28]. Mice (*n* = 6) were treated with vehicle (10% DMSO; 10 mL/kg; per os (p.o.); negative control), ASA (100 mg/kg; p.o.; positive control), or MECN (100, 250, and 500 mg/kg; p.o.) for 60 min before the administration of phlogistic agent (0.6% acetic acid; 10 mL/kg; intraperitoneal (i.p.)). The animals were then immediately placed individually in glass cages and 5 min later abdominal constriction resulting from acetic acid injection involving contraction of the abdomen and stretching of at least one hind limb was measured. The number of abdominal constrictions produced was counted cumulatively for 25 min. Antinociceptive activity was expressed as the reduction of the mean number of abdominal constrictions in test groups compared to the control group, calculated as the percentage inhibition of abdominal constrictions (percentage of inhibition) using the following formula: (mean [(control − test group)/control group] × 100%).

### 2.9. Hot Plate Test

The hot plate test was carried out according to the method previously described [29]. Mice (*n* = 6) were placed on a hot plate (Model 7280; Ugo Basile, Milan, Italy) heated to 50 ± 0.2°C, and the latency to a discomfort reaction was recorded when the animals licked their forepaws or hind paws or jumped. Animals were selected a day prior to the test based on their reactivity: only animals with response latencies of 5–7 sec were used. The discomfort reaction time was recorded before and at 60, 90, 120, 150, 180, and 210 min following the administration of vehicle (10 mL/kg; p.o.; positive control), morphine (5 mg/kg; i.p.), or MECN (100, 250, and 500 mg/kg; p.o.) 60 min before the test. A cut-off time of 20 sec was set to prevent tissue injury. Prolongation of the latency times of the test groups compared with that of the controls, which indicates antinociceptive activity, was used for statistical comparison.

### 2.10. Formalin-Induced Paw Licking Test

The formalin-induced paw licking test was performed as previously described [30]. Rats (*n* = 6) received vehicle (10 mL/kg; p.o.), ASA (100 mg/kg; p.o.), morphine (5 mg/kg; i.p.), or MECN (100, 250, and 500 mg/kg; p.o.) 60 min before the formalin injection. Nociception was induced by injecting 50 *μ*L formalin (5% v/v) in the intraplantar (i.pl.) region of the right hind paw. Following injection of the phlogistic agent formalin, the animals were immediately placed individually in a transparent observation glass chamber. The duration the animal spent licking the injected paw (considered an indicator of pain) was recorded. The nociceptive response develops in two phases: 0–5 min after formalin injection (early phase, neurogenic pain response) and 15–30 min after formalin injection (late phase, inflammatory pain response), which were recorded.

### 2.11. Involvement of Opioidergic System

The protocol used was similar to the method previously described [31]. To evaluate the involvement of opioidergic system in the antinociceptive properties of MECN, separate groups of animals (*n* = 6) were treated with the nonselective opioid receptor antagonist naloxone (5 mg/kg; i.p.) 15 min before the administration of vehicle (10 mL/kg; p.o.) or MECN (500 mg/kg; p.o.). The antinociceptive effect was evaluated using the acetic acid-induced abdominal writhing test, hot plate test, and formalin-induced paw licking test as described above.

### 2.12. Involvement of l-Arg/Nitric Oxide/Cyclic Guanosine Monophosphate Pathway

To investigate the possible contribution of l-arg/nitric oxide/cyclic guanosine monophosphate (l-arg/NO/cGMP) pathway to the antinociceptive effect of MECN, the previously described method was adopted [28]. Mice (*n* = 6) were pretreated with the NO precursor, l-arg (20 mg/kg; i.p.), the NO inhibitor, l-NAME (20 mg/kg; i.p.), the nonspecific guanylyl cyclase inhibitor, ODQ (2 mg/kg; i.p.), or combinations thereof (l-arg + l-NAME or l-arg + ODQ) 5 min before the administration of vehicle (10 mL/kg; p.o.) or MECN (500 mg/kg; p.o.). Sixty minutes after the administration of test solutions, mice were subjected to the acetic acid-induced abdominal writhing test as described earlier.

### 2.13. Statistical Analysis

Statistical analysis was performed using GraphPad Prism version 6.04 for Windows (GraphPad Software, San Diego, CA, USA). Data are expressed as the mean ± standard error of the mean (SEM). Mean differences between the control and treatment groups were determined using one-way analysis of variance (ANOVA) followed by Tukey's* post hoc* test. In all cases, differences were considered significant if *p* < 0.05.

## 3. Results

### 3.1. Phytochemicals Constituents of MECN

Except for alkaloids and tannins, the phytochemicals screening of MECN showed the presence of flavonoids, saponins, steroids, and triterpenes.

### 3.2. UHPLC Profile of MECN

#### 3.2.1. Identification of Phenolic Compounds in MECN

**Figure 1:**
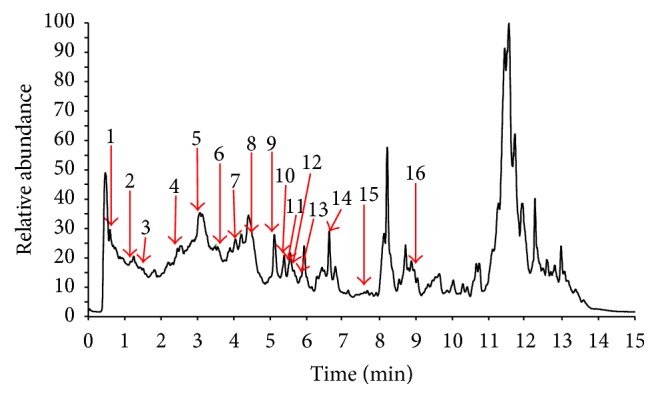
TIC (Total Ion Chromatography) profile of UHPLC-ESI of* C. nutans* extract. The numbering peaks correspond to those listed in Table 1.

*C. nutans* extract was analyzed based on the accurate mass data of the molecular ions, in which ions detected were tentatively identified by their generated molecular formula using the data analysis software (Xcalibur) that provided list of possible elemental formulas. These findings were compared together with the standard flavonoids available in the laboratory and further supported by the thorough survey of the literature (Figure 1). The widely accepted accuracy threshold for confirmation of elemental compositions was established at 5 ppm.

In the present study, major flavonoid compounds found in MECN belonged to the family of flavone* C*-glycoside. The UHPLC-ESI analysis of MECN revealed the presence of 16 phenolic compounds (Table 1). The compounds detected were gallic acid, 4-hydroxybenzoic acid, caffeic acid, coumaric acid, ferulic acid, schaftoside, vitexin, orientin, isoorientin, isovitexin, luteolin, apigenin, forsythosides H, forsythosides I, diosmetin glycoside, and diosmetin.

### 3.3. GC-MS Profile of MECN

**Figure 2:**
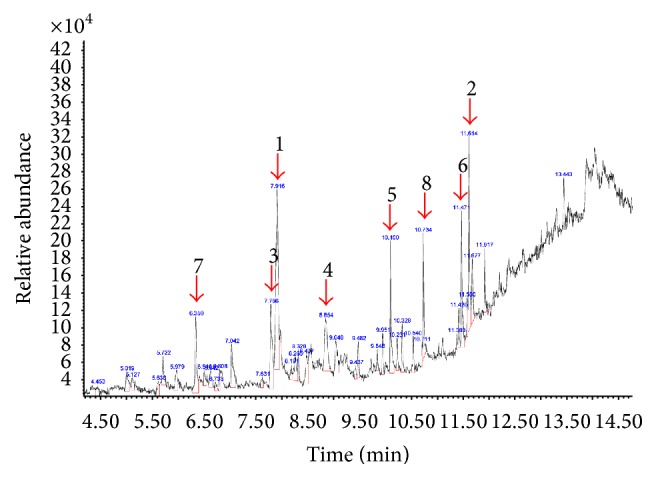
GC-MS profile of MECN showing approximately 39 detected peaks with major peaks representing (i) 2-ethyl-oxetane (16.6%), (ii) 9,12,15-octadecatrienoic acid (7.6%), (iii) 2,3-dimethylpyridine (6.4%), (iv) 3-deoxy-d-mannoic lactone (5.7%), (v) neophytadiene (5.4%), (vi) phytol (5.3%), (vii) 2,3-dihydrobenzofuran (4.5%), and (viii)* n*-hexadecanoic acid (4.6%).

The GC-MS profile of MECN is shown in Figure 2. A total of 39 peaks were identified from MECN with the major compounds constituted of (i) 2-ethyl-oxetane (16.6%), (ii) 9,12,15-octadecatrienoic acid (7.6%), (iii) 2,3-dimethylpyridine (6.4%), (iv) 3-deoxy-d-mannoic lactone (5.7%), (v) neophytadiene (5.4%), (vi) phytol (5.3%), (vii) 2,3-dihydrobenzofuran (4.5%), and (viii)* n*-hexadecanoic acid (4.6%).

### 3.4. Acetic Acid-Induced Abdominal Writhing Test

**Figure 3:**
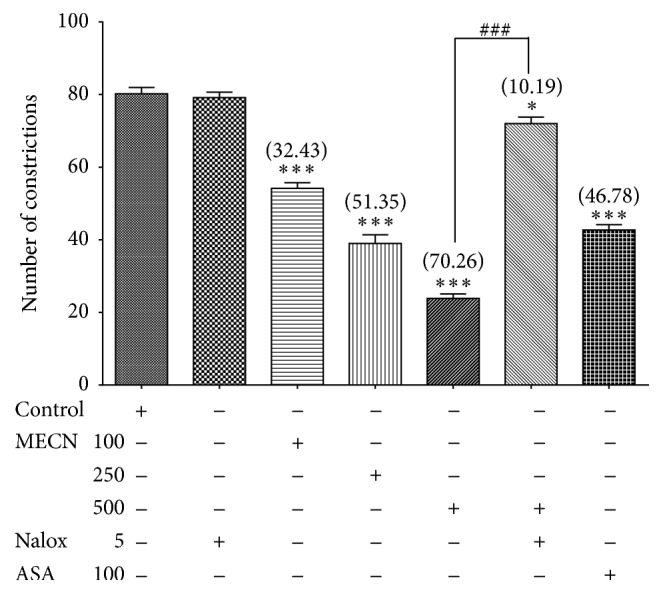
Effect of MECN on acetic acid-induced abdominal constriction in mice. Animals were treated with vehicle (10 mL/kg, p.o.), ASA (100 mg/kg, p.o.), or MECN (100, 250, and 500 mg/kg, p.o.) 60 min before acetic acid (0.6%, 10 mL/kg, i.p.) treatment. Naloxone (Nalox, 5 mg/kg, i.p.) was administered 15 min before MECN (500 mg/kg, p.o.) or vehicle (10 mL/kg, p.o.). Each column represents the mean ± SEM of six mice. Statistical analyses were performed using 1-way ANOVA followed by Tukey's* post hoc* test. ^*∗*^ *p* < 0.05, ^*∗∗∗*^ *p* < 0.001 compared to control group; ^###^ *p* < 0.001 compared to 500 mg/kg MECN-treated group. Values in parentheses denote percentage of inhibition.

Figure 3 depicts the effect of MECN on acetic acid-induced abdominal writhing in mice. Administration of MECN (100, 250, and 500 mg/kg)* per os* produced significant (*p* < 0.001) and dose-related inhibition in the number of acetic acid-induced abdominal writhing responses. At the tested doses, MECN produced 32.43, 51.35, and 70.26% inhibition of constrictions, respectively, in comparison to the control group. The ED_50_ value recorded for the abdominal constriction test was 279.3 mg/kg. ASA, a standard nonsteroidal anti-inflammatory drug (NSAID), also caused a significant inhibition (46.78%) of acetic acid-induced abdominal writhing, which is equal in strength to the 250 mg/kg MECN.

### 3.5. Hot Plate Test

The antinociceptive effect of orally administered MECN against thermal-induced nociception is described in Table 2. At 100 and 250 mg/kg, MECN caused no significant changes in response latency to thermal-induced nociception when compared to the control group. In contrast, 500 mg/kg MECN significantly (*p* < 0.05) delayed response latency at the interval of 60 to 210 min after its administration as compared to the control group. Moreover, the opioid agonist, morphine, caused dose-dependent prolongation of latency response time at the interval of 60 to 210 min as compared to the control group (Table 2).

### 3.6. Formalin-Induced Paw Licking Test

**Figure 4:**
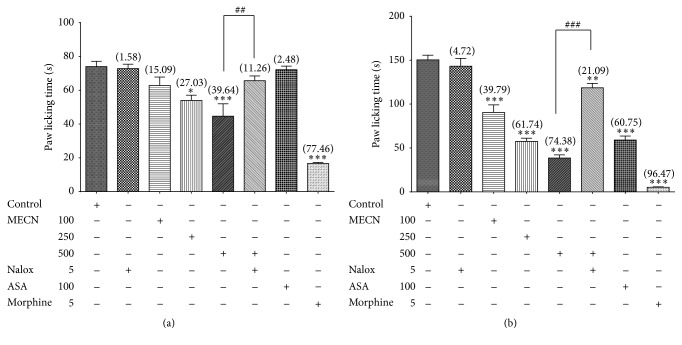
Effect of MECN on formalin-induced paw licking in rats. (a) Early phase; (b) late phase. Rats were treated with vehicle (10 mL/kg, p.o.), ASA (100 mg/kg, p.o.), MECN (100, 250, and 500 mg/kg, p.o.), or morphine (5 mg/kg, p.o.) 60 min before intraplantar administration of 5% formalin (50 *μ*L in distilled water) into the right hind paw. Naloxone (Nalox, 5 mg/kg, i.p.) was administered 15 min before MECN (500 mg/kg, p.o.) or vehicle (10 mL/kg, p.o.). Each column represents the mean ± SEM of six rats. Statistical analyses were performed using 1-way ANOVA followed by Tukey's* post hoc* test. ^*∗*^ *p* < 0.05, ^*∗∗*^ *p* < 0.01, and ^*∗∗∗*^ *p* < 0.001 compared to control group; ^##^ *p* < 0.01, ^###^ *p* < 0.001 compared to 500 mg/kg MECN-treated group. Values in parentheses denote percentage of inhibition.

Figure 4 shows the antinociceptive activity of orally administered MECN when assessed using the formalin-induced paw licking test. The extract, at 250 and 500 mg/kg, caused a significant (*p* < 0.05) decrease in the formalin-induced licking time in the first phase (neurogenic phase; 0–5 min; Figure 4(a)) of the test with the recorded percentage of nociceptive inhibition of 27.03% and 39.64%, respectively. In the second phase (inflammatory phase; 15–30 min; Figure 4(b)) of the test, all doses of MECN decreased the formalin-induced licking time significantly (*p* < 0.05) with the recorded percentage of antinociception ranging between 40 and 74% when compared to the control group. Thus, the recorded ED_50_ value for the early and late phases was >500 mg/kg or 227.7 mg/kg, respectively. Standard drugs, ASA, decreased the licking time significantly (*p* < 0.05) (60.75%) only in the second phase while morphine caused significant (*p* < 0.05) inhibition of the pain response in both phases of formalin test (77.46% and 96.47%, resp.).

### 3.7. Opioidergic System Involvement

Figure 3, Table 2, and Figures 4(a) and 4(b) depict the involvement of opioid receptors in the antinociceptive effect of MECN assessed using the abdominal constriction-, hot plate-, and formalin-induced paw licking test, respectively. The extract was prechallenged with a nonselective opioid antagonist, naloxone, prior to assessment using various nociceptive models. Used alone, naloxone did not affect acetic acid-induced nociception, whereas pretreatment with naloxone significantly reversed (*p* < 0.001) the antinociceptive effect of MECN.

In the hot plate test, naloxone alone also did not cause any significant changes in the response latency at 60, 90, 120, 150, 180, or 210 min whereas pretreatment with naloxone significantly (*p* < 0.05) blocked the antinociceptive effect of MECN at 60, 90, 120, 150, 180, and 210 min. Naloxone also reversed the antinociceptive effect of opioid agonist, morphine, significantly (*p* < 0.05) at 60, 90, 120, 150, 180, and 210 min.

Moreover, the antinociceptive effect of MECN and morphine in both phases of the formalin test was significantly antagonized at the early phase (*p* < 0.01) and late phase (*p* < 0.001) after pretreatment with naloxone.

### 3.8. l-Arg/NO/cGMP Pathway Involvement

**Figure 5:**
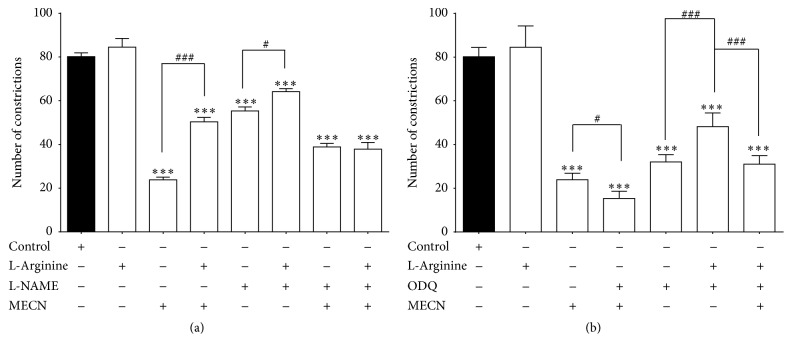
The involvement of l-arg/NO/cGMP pathway in the modulation of MECN antinociception as assessed using the abdominal constriction test. (a) Effects of pretreating animals with l-arg, l-NAME, or their combination on the antinociceptive activity of MECN. (b) Effects of l-arg, ODQ, or their combination on the antinociceptive activity of MECN. Animals were treated with MECN (500 mg/kg, p.o.) or vehicle (10 mL/kg, p.o.) 60 min before acetic acid (0.6%, 10 mL/kg, i.p.) treatment. l-arg (20 mg/kg, i.p.), l-NAME (20 mg/kg, i.p.), ODQ (2 mg/kg, i.p.), or combinations thereof (l-arg + l-NAME or l-arg + ODQ) were administered 5 min before MECN (500 mg/kg, p.o.) or vehicle (10 mL/kg, p.o.). Each column represents the mean ± SEM of six mice. Statistical analyses were performed using 1-way ANOVA followed by Tukey's* post hoc* test. ^*∗∗∗*^ *p* < 0.001 compared to control group; ^#^ *p* < 0.05, ^###^ *p* < 0.001 compared to 500 mg/kg MECN, l-NAME, l-arg, l-arg + l-NAME, or l-arg + ODQ group.

Figures 5(a) and 5(b) show the effect of l-arg, l-NAME, ODQ, or combinations thereof on antinociceptive activity of MECN assessed using the acetic acid-induced abdominal constriction test. l-arg did not affect the acetic acid-induced nociception in 10% DMSO-treated group but significantly (*p* < 0.05) reversed the antinociceptive activity of MECN. Conversely, l-NAME caused significant (*p* < 0.05) reduction in the acetic acid-induced nociception in 10% DMSO-treated group and significantly (*p* < 0.05) reversed the antinociceptive activity of MECN. On the other hand, pretreatment of a combination between l-arg and l-NAME (as l-arg + l-NAME) exerted significant (*p* < 0.05) antinociceptive activity in the 10% DMSO-treated group but significantly (*p* < 0.05) reversed the antinociceptive activity of MECN.

Pretreatment with ODQ or a combination between l-arg and ODQ (as l-arg + ODQ) significantly (*p* < 0.05) attenuated the acetic acid-induced nociception in 10% DMSO-treated group but failed to significantly affect the antinociceptive activity of MECN.

## 4. Discussion

The extract (MECN) demonstrated a wide safety margin and is safe for oral consumption up to the dose of 5000 mg/kg while for the chronic oral consumption the dose is up to 2500 mg/kg, all of which did not cause any toxicity, mortality, or body weight changes. From the acute and subchronic toxicities study, the dose range (100, 250, and 500 mg/kg) for antinociceptive study was determined and decided to be 10-, 20-, and 50-fold reduction of the dose used in acute toxicity study (5000 mg/kg) [32].

Phytochemical screening of MECN revealed the presence of flavonoids, saponins, triterpenes, and steroids, which is in line with previous reports [11, 19, 23, 33]. The UHPLC profiling of MECN demonstrated the presence of several flavonoid-based compounds that belong to the family of flavone* C*-glycoside as reported previously by Chelyn et al. [34]. Sixteen compounds were detected in MECN, namely, gallic acid, 4-hydroxybenzoic acid, caffeic acid, coumaric acid, ferulic acid, schaftoside, vitexin, orientin, isoorientin, danisovitexin, luteolin, apigenin, forsythosides H, forsythosides I, diosmetinacetylglycoside, and diosmetin. Although MECN as a crude extract contains various types of bioactive compounds, flavonoid-based compounds, in part, have been reported to demonstrate antinociceptive activity. Of those detected compounds, at least gallic acid [35], caffeic acid [36], ferulic acid [37], vitexin [38], and apigenin [39] have been reported to exert antinociceptive activity when given orally. These compounds are suggested to work synergistically to exert the observed antinociceptive activity in MECN.

Following the antinociceptive studies, MECN attenuated the chemical-induced (i.e., acetic acid- and formalin-induced) and thermal-induced (i.e., hot plate model) nociceptive models suggesting that the antinociceptive profile of MECN includes peripheral and central mechanisms of action. This suggestion was based on previous claims that any substances that can attenuate the abdominal constriction and hot plate tests [40] or reversed the response latency to formalin-induced nociception in both the early and late phases of formalin test [41] possess peripheral and central antinociceptive activity.

Further postulations could also be made regarding the mechanisms of antinociception exerted by MECN based on the extract ability to attenuate the respective nociceptive model. The abdominal constriction test is a characteristic model for inflammatory pain and is frequently used to investigate the antinociceptive potential of any extracts or natural/synthetic compounds [31]. Positive results obtained from this model also, if not supported by other models, could suggest that the tested extract/compound possesses peripherally mediated antinociceptive activity [42]. According to Ikeda et al. [43], increased level of inflammatory mediators (i.e., cyclooxygenase (COX), prostaglandins (PGs), histamine, serotonin, bradykinin, etc.) upon the administration of acetic acid leads to the excitation of peripheral nociceptive neurons entering dorsal horn of the central nervous system. Therefore, the ability of MECN to attenuate acetic acid-induced nociception indicates the peripheral antinociceptive action partly via the attenuation of several inflammatory mediators' action that lead to impediment of pain transduction at the primary afferent nociceptors. Although considered a sensitive nociception model, this test is also believed to be a nonspecific test as muscle relaxants and other drugs might give false positive results [44].

To avoid misinterpretation of results obtained from the abdominal constriction model, additional experiments using other models of nociception are warranted. The hot plate test is aimed at studying the spinal antinociceptive potential of any tested substances by measuring the animal nociceptive response latencies to thermal stimulus following treatment with the substances. The principal response of thermal-induced nociception occurs predominantly at the supraspinal level [41]. The hot plate test is specifically used to investigate the central antinociceptive potential of any extract/compound and is specifically affected only by the centrally acting drugs (i.e., opioids) [44]. The ability of MECN to reverse the painful thermal stimulus suggests the involvement of central antinociceptive mechanism. However, the fact that highest dose of MECN is required to attenuate thermal-induced nociception indicates that MECN was not a strong agent at the central thermal-stimulated nociceptive pathway.

To further support the suggested involvement of peripheral and central mechanisms in the modulation of antinociceptive activity of MECN, the formalin-induced paw licking test (or formalin test) was adopted. This model can be used to investigate the ability of new extract/compound to affect the peripheral or central nociceptive pathways due to its characteristic biphasic nociception, known as early phase and late phase [24]. The former corresponds to neurogenic pain, is observed immediately after the administration of formalin, and persists for 5 min (0–5 min) as a response to the direct action of formalin on nociceptors in the subplantar region. The late phase corresponds to inflammatory-mediated pain resulting from a tonic response due to the release of inflammatory mediators [24]. The late phase occurs between 15 and 30 min after the administration of formalin. Moreover, the ability to reverse the early phase suggests the extract/compound ability to inhibit the non-inflammatory-mediated nociception while the ability to reverse the late phase suggests the extract potential to inhibit the inflammatory-mediated nociception. From the results obtained from the three models of nociception, MECN is suggested (i) to have peripheral and central antinociceptive action; (ii) to possess antinociceptive activity against both the non-inflammatory-mediated and inflammatory-mediated nociception; and (iii) to exert opioids' characteristic due to its ability to attenuate the peripheral and central models of nociception.

Being the standard drugs for the treatment of pain, opioids effectiveness has been overshadowed by various side effects including dependence and tolerance. In an attempt to find better pain-relieving agents with possibly no or less side effects, the potential of MECN to exert its antinociceptive activity via the opioid receptors was also investigated using the three nociceptive models. From the results obtained, the peripheral and central antinociceptive activities of MECN were blocked by naloxone, a nonselective opioid antagonist, suggesting the involvement of opioid receptor system.

Further study on the involvement of l-arg/NO/cGMP pathway in the MECN-induced antinociceptive effect was also carried out based on previous reports that standard analgesics like morphine also utilized this pathway to exert its analgesic effect. To the best of our knowledge, there has been no attempt to determine the role of l-arg/NO/cGMP pathway in the modulation of antinociceptive activity of MECN. NO production in the body leads to the activation of soluble guanylate cyclase (sGC) and elevation in the cGMP level within the target cells [45]. Despite the various roles played by NO, its involvement in the mechanisms of pain modulation, either as an antinociceptive or as a pronociceptive agent, is well acknowledged and has been attributed to the NO capability to manipulate nociception processing in both the peripheral and central nervous systems [45, 46]. The l-arg/NO/cGMP pathway has been reported to play significant role in the modulation of antinociceptive activity of morphine [46, 47]. Since MECN was shown to possess characteristics of morphine, there is a need to also determine the role of l-arg/NO/cGMP pathway in the antinociceptive activity of MECN. From the results obtained, the presence of NO from the conversion of l-arg did not affect nociception threshold at the respective dose of l-arg used but reduced the antinociceptive intensity of MECN indicating the importance of NO presence. While reduction of NO level due to the administration of l-NAME alone, at the respective dose used, triggered antinociceptive action, it also reversed the antinociceptive activity of MECN. The observations following the administration of l-NAME as described above plausibly suggest that although decrease in NO level triggered antinociception as previously reported, reduced NO did not synergistically enhance or maintain, but reduced, the antinociceptive intensity of MECN. The reason for this observation was not clearly understood, but it is suggested that, at certain concentration of NO reduction, MECN tends to reduce, but not lose, its activity. The ability to maintain the antinociceptive activity also possibly suggested that MECN, which contains several bioactive compounds that exert antinociceptive activity, triggered several antinociceptive mechanisms other than the NO-mediated pathway. NO also increases cGMP levels by activating soluble guanylyl cyclase (sGC), which affects pain and analgesia. The ability of cGMP pathway to affect nociceptive process [48] can be seen when ODQ, which inhibits the cGMP pathway, induced antinociceptive activity when given alone. However, ODQ failed to affect the antinociceptive activity of MECN suggesting that MECN might have triggered an NO-mediated, cGMP-independent pathway. The role of NO-dependent, cGMP-independent pathway in the modulation of antinociceptive activity has been reported elsewhere [49] and might support the present observations. Overall, these observations suggest that the antinociceptive activity of MECN involves the modulation of, partly, l-arg/NO-mediated, but cGMP-independent, pathway. Moreover, based on these observations, the antinociceptive activity of MECN is suggested to involve modulation of different subsets of nociceptive primary sensory neurons.

## 5. Conclusions

This is the first demonstration that oral systemic administration of MECN has both central and peripheral antinociceptive activities, which occur via the activation of opioid receptors and modulation of the l-arg/NO-mediated, but cGMP-independent, pathway.

## Figures and Tables

**Table 1:** Phenolic compounds tentatively identified in *C. nutans* extract.

Peak number	*t*_*R*_ (min)	[M-H]^−^ (*m/z*)	Error (ppm)	Molecule formula	Proposed compound
1	0.63	169.01270	−2.661	C_7_H_5_O_5_	Gallic acid
2	1.22	137.02347	1.091	C_7_H_5_O_3_	4-Hydroxybenzoic acid
3	1.51	179.03314	−4.162	C_9_H_7_O_4_	Caffeic acid
4	2.55	163.0387	−1.598	C_9_H_7_O_3_	Coumaric acid
5	3.06	563.13776	−3.253	C_26_H_27_O_14_	Schaftoside
6	3.51	193.04881	−3.757	C_10_H_9_O_4_	Ferulic acid
7	4.04	431.09622	−2.443	C_21_H_19_O_10_	Vitexin
8	4.52	447.09158	−1.359	C_21_H_19_O_11_	Orientin
9	5.11	623.19568	−2.193	C_29_H_35_O_15_	Forsythoside H
10	5.37	447.09152	−1.158	C_21_H_19_O_11_	Isoorientin
11	5.50	623.19604	−2.193	C_29_H_35_O_15_	Forsythoside I
12	5.55	431.09644	−1.933	C_21_H_19_O_10_	Isovitexin
13	5.70	503.11783	−1.138	C_24_H_24_O_12_	Diosmetin glycoside
14	6.85	285.03860	−2.682	C_15_H_9_O_6_	Luteolin
15	7.66	269.04404	−1.523	C_15_H_9_O_5_	Apigenin

**Table 2:** Effects of MECN on the hot plate test in mice.

Group	Dose (mg/kg)	Latency of discomfort(s) at respective time interval (min)						
		0 min	60 min	90 min	120 min	150 min	180 min	210 min
10% DMSO		6.29 ± 0.15	6.88 ± 0.29	6.89 ± 0.31	6.28 ± 0.12	6.76 ± 0.43	6.67 ± 0.33	6.46 ± 0.12
Nalox	5	6.55 ± 0.33	6.02 ± 0.34	5.50 ± 0.29	5.53 ± 0.37	5.63 ± 0.09	5.35 ± 0.15	5.20 ± 0.39
MECN	100	6.52 ± 0.24	6.50 ± 0.33	6.23 ± 0.25	6.25 ± 0.21	6.32 ± 0.27	5.99 ± 0.26	6.17 ± 0.22
	250	6.08 ± 0.11	6.28 ± 0.28	6.68 ± 0.22	6.78 ± 0.19	6.59 ± 0.32	6.17 ± 0.18	6.39 ± 0.20
	500	6.65 ± 0.35	10.28 ± 0.81^*∗∗∗*^	9.92 ± 0.55^*∗∗*^	9.52 ± 1.08^*∗∗∗*^	9.14 ± 0.51^*∗*^	9.14 ± 0.36^*∗*^	8.78 ± 0.81^*∗*^
Nalox + MECN	5 + 500	6.60 ± 0.38	5.98 ± 0.38^#^	5.52 ± 0.57^#^	5.79 ± 0.27^#^	5.54 ± 0.30^#^	5.56 ± 0.32^#^	5.59 ± 0.32^#^
Morphine	5	6.02 ± 0.15	17.00 ± 0.90^*∗∗∗*^	18.42 ± 0.47^*∗∗∗*^	17.25 ± 0.93^*∗∗∗*^	13.47 ± 1.31^*∗∗∗*^	11.87 ± 1.04^*∗∗∗*^	11.15 ± 0.71^*∗∗∗*^
Morphine + Nalox	5 + 5	6.58 ± 0.24	7.08 ± 0.24^#^	7.40 ± 0.21^#^	7.58 ± 0.40^#^	7.03 ± 0.36^#^	7.33 ± 0.40^#^	7.23 ± 0.40^#^

Mice were treated with vehicle (10 mL/kg, p.o.), MECN (100, 250, and 500 mg/kg, p.o.), or morphine (5 mg/kg, p.o.) 60 min before the test. Naloxone (Nalox, 5 mg/kg, i.p.) was administered 15 min before MECN (500 mg/kg, p.o.), morphine (5 mg/kg, p.o.), or vehicle (10 mL/kg, p.o.). Data expressed are the mean ± SEM of reaction time (sec) of six mice. Statistical analysis was performed using 2-way ANOVA followed by Tukey's *post hoc* test. ^*∗*^ *p* < 0.05, ^*∗∗*^ *p* < 0.001, and ^*∗∗∗*^ *p* < 0.0001 compared to control; ^#^ *p* < 0.0001 compared to 500 mg/kg MECN or morphine-treated group.
